# Sex differences in mortality and hospitalization in heart failure with preserved and mid-range ejection fraction: a systematic review and meta-analysis of cohort studies

**DOI:** 10.3389/fcvm.2023.1257335

**Published:** 2024-01-05

**Authors:** You Deng, Jun Zhang, Jitao Ling, Qingwen Hu, Tianggang Song, Yi Xu, Menglu Liu, Yuting Wu, Kaibo Mei, Jiawei Chen, Huilei Zhao, Xiao Liu

**Affiliations:** ^1^ Department of Cardiology, The Third People’s Hospital of Pingxiang, Pingxiang, Jiangxi, China; ^2^ Department of Cardiology, Jiujiang NO.1 people's Hospital, Jiujiang, Jiangxi, China; ^3^ Department of Endocrinology and Metabolism, The Second Affiliated Hospital, Jiangxi Medical College, Nanchang University, Nanchang, Jiangxi, China; ^4^ Department of Cardiology, Seventh People’s Hospital of Zhengzhou, Zhengzhou, Henan, China; ^5^ Department of Anesthesiology, The Third Hospital of Nanchang, Nanchang, Jiangxi, China; ^6^ Department of Cardiology, Sun Yat-sen Memorial Hospital, Guangzhou, Guangdong, China

**Keywords:** sex, HFpEF, HFmrEF, HF, prognosis, meta-analysis

## Abstract

**Introduction:**

The influence of sex on the prognosis of heart failure with preserved or intermediate ejection fraction (HFpEF and HFmrEF) remains uncertain. This study aimed to investigate whether sex differences impact the prognosis of patients diagnosed with HFpEF and HFmrEF.

**Methods:**

A comprehensive search across three databases (PubMed, the Cochrane Library, and Embase) was conducted to identify sex-related prognostic cohort studies focusing on HFpEF and HFmrEF. Risk estimates were synthesized using the random effects model. The analysis included 14 cohorts comprising 41,508 HFpEF patients (44.65% males) and 10,692 HFmrEF patients (61.79% males).

**Results:**

Among HFpEF patients, men exhibited significantly higher rates of all-cause mortality (13 studies; hazard ratio (HR): 1.24, 95% confidence interval (CI): 1.15 to 1.33)) and cardiovascular disease mortality (5 studies; HR: 1.22, 95% CI: 1.14 to 1.31) compared to women. However, no significant difference was observed in HF admissions. For HFmrEF patients, men displayed notably higher all-cause mortality (HR: 1.21, 95% CI: 1.12 to 1.31) but no significant differences in cardiovascular mortality or HF admissions.

**Discussion:**

These findings suggest that male patients diagnosed with HFpEF and HFmrEF may face a more unfavorable prognosis in terms of all-cause mortality. Variations were noted in cardiovascular mortality and HF admissions, indicating potential complexities in sex-related prognostic factors within these heart failure categories. In summary, male patients with HFpEF and HFmrEF may have a more unfavorable prognosis.

## Graphical Abstract

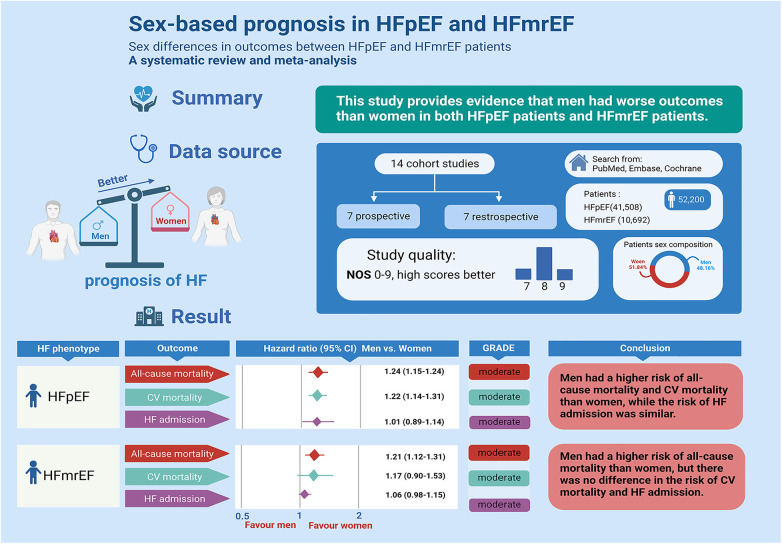

## 1 Introduction

There are approximately 64 million people in the world with heart failure (HF), and more than half of them are women (1–3). With economic development and the acceleration of population aging, the incidence of global HF is still increasing (3, 4). The latest HF guidelines classify HF by ejection fraction (5): HF with reduced ejection fraction (HFrEF; EF = <40%), HF with mid-range EF (HFmrEF; EF: 41%–49%), and HF with preserved EF (HFpEF; EF ≥ 50%) (6, 7). More than half of HF cases are HFpEF and HFmrEF, with an increasing trend in recent years (2). Epidemiological studies provide evidence that sex influences the outcomes of HFrEF patients, particularly with regard to men, who exhibit higher all-cause mortality rates compared to women (5, 8, 9). However, the impact of sex differences on the prognosis of patients with HFpEF or HFmrEF is a topic that lacks clarity in the existing literature. Therefore, further research is needed to comprehensively understand the relationship between sex differences and prognosis in both HFpEF and HFmrEF. We reviewed the literatures to elucidate whether sex differences influence the prognosis of patients with HFpEF or HFmrEF.

## 2 Methods

### 2.1 Protocol registration and search strategy

This meta-analysis was registered with PROSPERO (International Prospective Register of Systematic Reviews. -registration number CRD42022349968) and reported according to the Preferred Reporting Items for Systematic Reviews and Meta-Analyses (PRISMA) guidelines (Supplementary Table S1) (10).

Two authors (J.W-C. and X-L) independently carried out the database search, selection, extraction, and analysis of data. As of July 2022, we searched three databases, including PubMed, the Cochrane Library, and Embase, for all literature related to the topic. No language was restricted. All searches used the following search terms: (“sex” OR “sex”) AND (“Heart failure” OR “Heart failure with mid-range ejection fraction” OR “Heart failure with preserved ejection fraction”). Specific search strategies are shown in Supplementary Table S2. In addition, bibliographies and conference abstracts of related literature were searched for additional relevant articles.

### 2.2 Selection criteria and study selection

The criteria included in this study were as follows: (1) participant type: patients (age > 18 years) who were diagnosed with HFpEF or HFmrEF; (2) exposure and comparator: men vs. women; (3) outcomes: mortality [all-cause and cardiovascular diseases (CVDs)], HF admission, and all-cause admission; and (4) types of studies: observational cohort studies or *post hoc* analyses of clinical trials.

Studies with the following criteria were excluded: (1) no studies reported multivariate adjusted results. (2) articles with insufficient data (reviews, editorials, preclinical studies, practice guidelines, comments); and (3) studies with irrelevant purposes of this meta-analysis.

We imported all the literature preliminarily retrieved into management software (Endnote X9.2 software, Thomson Reuters, New York, NY). Then, we manually and automatically removed the duplicate literature and eliminated the remaining literature by reading the title and abstract. Finally, after preliminary screening, the complete literature that may meet the requirements was obtained. If there were any inconsistencies in the retrieval process, we resolved them through discussion (X.L.) to reach a consensus.

### 2.3 Data collection and quality assessment

We collected the following information by the predefined requirements for inclusion: study characteristics (first author's name, year of publication, region, origin of patients, type of design, and mean follow-up time), patient characteristics (sample size, age, sex, HF phenotype, and definition), and outcomes (adjusted hazard ratios (HRs), the corresponding 95% confidence interval (CI), and adjustments). Study quality was determined using the Newcastle‒Ottawa Quality Scale (NOS) (11).

### 2.4 Statistical analysis

To elucidate the relationship between sex differences and prognosis in HFpEF and HFmrEF patients, we pooled the adjusted HRs with 95% CIs and used the inverse-variance method. We assessed the heterogeneity across the included articles using Cochrane's *Q* test (*P *< 0.1 marks significant). The inconsistency was assessed by the *I*^2^ test (30%–50%: low, 50%–75%: moderate, >75%: high) (12). We used a random effects model due to potential heterogeneity within observational studies.

Subgroup analysis would be performed when the number of studies used for outcomes is greater than 10. Subgroup analysis was stratified according to the following factors: study design, sample size, region, mean follow-up time, and adjustment. According to the guidelines, when the number of studies included was more than 10, publication bias was evaluated by funnel plots, Egger's test, and Begg's test (13). Graphic abstracts and mechanisms were created in the Biorender web-based tool. We used sensitivity analysis by omitting each study or excluding studies with HFpEF with a definition of ejection fraction not less than 50% to evaluate the robustness. Data analysis was processed by Stata software (Version 16.0, Stata Corp LP, College Station, Texas, USA). *P *< 0.05 indicated a significant difference, and all results were tested bilaterally.

### 2.5 Quality of evidence

We assessed the quality or certainty of each outcome using the Grading of Recommendations Assessment, Development and Evaluation (GRADE) (14, 15). The quality of evidence for each result was evaluated by two authors, who provided evidence profile tables from the GRADEpro GDT (Guideline Development Tool).

## 3 Results

### 3.1 Literature retrieval

**Figure 1:**
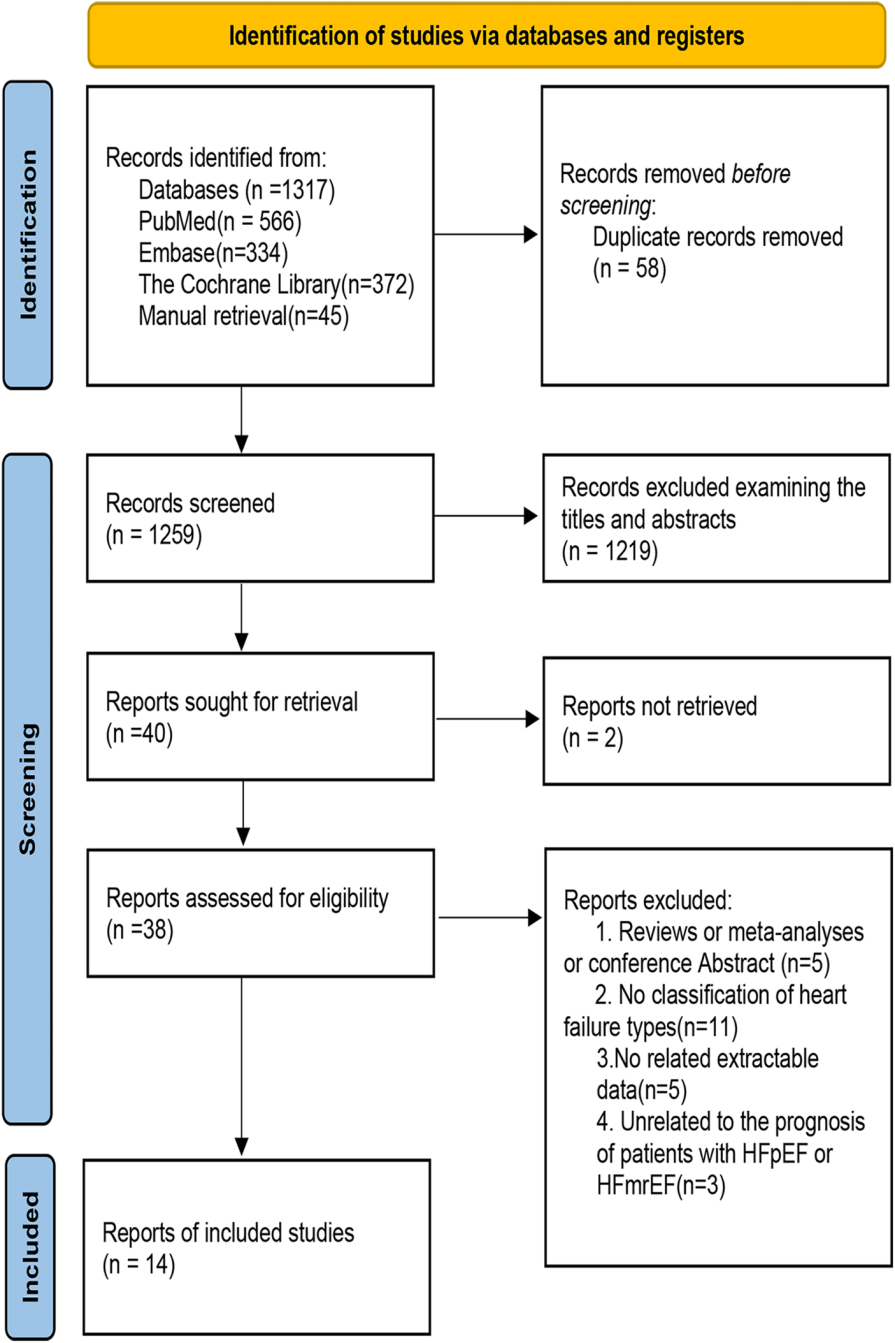
Flow chart of the study selection process in the meta-analysis of the association between sex differences and prognosis in patients with HFpEF and HFmrEF.

The whole retrieval process of the meta-analysis is shown in Figure 1. We retrieved 1,317 studies at the beginning, and then 40 studies were left by selecting titles and abstracts. Finally, 24 studies were further excluded after reviewing the full text. The specific elimination process was as follows: (1) nonoriginal research type literature, such as reviews (*n* = 5); (2) no classification of HF types (*n* = 11); (3) no related extractable data (*n* = 5); and (4) the outcomes of HFpEF and HFmrEF patients were not discussed (*n* = 3). As a result, we included 14 eligible studies (5, 8, 9, 16–26). Fourteen of the studies included data for HFpEF (5, 8, 9, 16–26) and three for HFmrEF (8, 9, 25). All excluded studies with the reasons (*n* = 24) are shown in Supplementary Table S3.

### 3.2 Study features and study quality

The main information of the 14 qualified studies is presented in Table 1. Of the 14 included cohort studies published between 2006 and 2021, 7 were prospective cohort studies, and 7 were retrospective cohort studies.

**Table 1:** Characteristics of included studies in this meta-analysis.

Author, year, region	Source of participants	Study design	Mean follow-up time(years)	HF phenotype, Definition	Sample of size/ Men (%)	Age		Outcome	Adjustments
						Male	Female		
Sakata et al. (8), Japan	CHART-2 study	Prospective cohort	6.3	HFpEF, ≥50%	3,193/ 64.80	68.3	71.6	All-cause mortality, CV mortality, HF admission	Age, height, weight, BMI, etiology of HF, clinical history (DM, hyperuricemia, smoking, AF, MI, hospitalization for HF, cancer), NYHA, DBP, heart rate, left ventricular diastolic dimension, LVEF, hemoglobin, albumin, eGFR, LDL-C, BNP, previous treatments (PCI, CABG, PMI), medications at baseline (antiplatelet, aldosterone antagonist, beta-blocker, ACE inhibitor and/or ARB, ACE inhibitor, diuretic)
				HFmrEF, 40%–49%	709/ 73.20	67.5	70.3	All-cause mortality, CV mortality, HF admission	
O'Meara et al. (5), US and Canada	CHARM	Retrospective cohort	3.17	HFpEF, >40%	3,023/ 59.91	NR	NR	All-cause mortality	Diabetic, BMI, NYHA class, smoking, bundle-branch block, cardiomegaly, prior HF hospitalization, DBP, diagnosis of chronic HF > 2 years, MI, dependent edema, heart rate, pulmonary crackles, pulmonary edema, mitral regurgitation, AF, rest dyspnea, and randomized treatment
Sharma et al. (22), US	4 US communities.	Retrospective cohort	1.0	HFpEF, ≥50%	8,987/ 35.08	75.6	78.1	All-cause mortality	Age, race, sex, HF hospitalization, HP, DM, AF, CHD, pulmonary HP, COPD, end-stage renal disease, heart rate, SBP, serum sodium, estimated glomerular filtration rate, BMI, and hemoglobin
Schmaltz et al. (21), Canada	APPROACH	Prospective cohort	>6.0	HFpEF, >50%	2,159/ 53.82	66.3	69.0	All-cause mortality	Age, indication for catheterization, comorbidities, extent of coronary disease, valvular disease, fine gradations of EF, medication and revascularization received
Stolfo et al. (9), Swedish	Swedish Heart Failure Registry	Retrospective cohort	>11.0	HFpEF, ≥50%	9,957/ 45.34	75.0	79.0	All-cause mortality, CV mortality, HF admission	Age, caregiver at Swede HF registration, specialty at Swede HF registration, follow-up referral specialty (physician specialty; not same as the HF nurse FUP), follow-up referral to outpatient HF nurse clinic, NYHA, BMI, blood pressure (SBP, DBP), heart rate, eGFR, NT-pro BNP, RAS inhibitors, MRA, digoxin, diuretic, nitrate, platelet inhibitor, oral anticoagulant, statin, beta-blocker, ICD and/or CRT, smoking, HP, DM, IHD, coronary revascularization, peripheral artery disease, severe bleeding, valve disease, anemia, cancer history, family type, education, income, number of children
				HFmrEF, 40%–49%	9,225/ 60.66	73.0	77.0	All-cause mortality, CV mortality, HF admission	
Chung et al. (17), Korean	Korean Heart Failure Registry	Retrospective cohort	3.07	HFpEF, ≥50%	764/ 35.47	67.3	71.1	All-cause mortality	Age, BMI, the prior history of HF, the etiology of HF, SBP, heart rate, the levels of blood hemoglobin, creatinine, sodium, and NT-pro BNP, the use of HF medications (including ACE inhibitor, ARB, beta-blockers, and spironolactone)
Blumer et al. (16), Asia, Europe, America	ASCEND-HF trial	Prospective cohort	0.49	HFpEF, >40%	1,018/ 49.12	72.0	76.0	All-cause mortality	Age, sex, race, geographic region, IHD, HF hospitalization, SBP, BUN, serum sodium, baseline dyspnea severity, paroxysmal nocturnal dyspnea, jugular venous distention, cerebrovascular disease, history of depression, and baseline medications (ACE inhibitor/ARB, beta-blocker, MRA).
Deswal et al. (18), US and Canada	National Heart, Lung, and Blood Institute	Retrospective cohort	3.25	HFpEF, ≥50%	719/ 52.57	67.0	70.0	All-cause mortality, HF admission	Age, SBP, heart rate, cardiothoracic ratio, EF, diabetes, HP, MI, GFR, etiology of HF, New York Heart Association (NYHA) class; number of signs or symptoms of HF, congestion on chest x-ray, dyspnea at rest or orthopnea, edema, rales, no. of signs or symptoms of HF, medications at baseline
Zsilinszka et al. (26), US	ADHERE-EM database	Retrospective cohort	0.49	HFpEF, ≥40%	4,161/ 32.52	79.9	82.4	All-cause mortality	Age, dyspnea at rest, edema, rales, elevated JVP, heart rate, SBP, oxygen saturation, congestion on x-ray, BUN, troponin I or T,BNP, creatinine, HP, smoking, diabetes, AF, COPD, prior HF, IV diuretics given, time to earliest IV diuretics, IV vasoactives/vasodilators given, time to earliest IV vasoactives/vasodilators, invasive procedures performed (eg, dialysis, mechanical ventilation, coronary artery bypass graft, catheterization, percutaneous transluminal coronary angioplasty), arrival by EMS, and loop diuretics, nitroglycerine, or morphine given by EMS.
Wang et al. (25) China	Tertiary referral hospitals in China	Prospective cohort	2.0	HFpEF, ≥50%	502/ 66.33	70.73	73.59	All-cause mortality, CV mortality, HF admission	Age, BMI, smoking, drinking, CHD, dilated cardiomyopathy, rheumatic cardiomyopathy, anema, hypoproteinemia
				HFmrEF, 40%–49%	758/ 64.91	69.37	72.80	All-cause mortality, CV mortality, HF admission	
Sotomi et al. (23), Osaka	PURSUIT- HFpEF study	Prospective cohort	1.09	HFpEF, ≥50%	870/ 44.71	79.75	82.23	All-cause mortality, HF admission	C- reactive protein, age, anemia (hemoglobin level <12 g/dl in women and <13 g/dl in men according to the World Health Organization definition15), HP, DM, dyslipidemia, CAD, chronic kidney disease, AF, obesity (BMI ≥ 25), cholinesterase level
Lam et al. (20), 25 countries	I-PRESERVE trial	Prospective cohort	4.125	HFpEF, ≥45%	4,128/ 39.66	71.0	72.0	All-cause mortality, CV mortality	Age, obesity, New York Heart Association status, HF cause, HF hospitalization, comorbidities/risk factors (HP, stable angina, MI, PCI/CABG, AF, diabetes, stroke/TIA, COPD/asthma, valve disease, smoking), EF capped at 60%, heart rate, SBP, hemoglobin, ln-NT-pro-BNP, natural log-neutrophil count, glomerular filtration rate capped at 90 mL/min per 1.73 m^2^, and all medications
Duca et al. (19), Vienna	Prospective national registry	Prospective cohort	2.5	HFpEF, ≥50%	260/ 30.38	72.0	73.0	CV mortality	Age, race, LVEF, AF, CAD, chronic obstructive pulmonary disease, HP, DM, BMI, heart rate, SBP, estimated glomerular filtration rate, NYHA.
Merrill et al. (24), US	TOPCAT trial	Retrospective cohort	3.3	HFpEF, ≥45%	1,767/ 50.08	71.0	72.1	All-cause mortality, HF admission	Age, race, LVEF, AF, CAD, chronic obstructive pulmonary disease, HP, DM, BMI, heart rate, SBP, estimated glomerular filtration rate, NYHA

CHART-2, Chronic Heart Failure Analysis and Registry in the Tohoku District-2; CHARM, Candesartan in Heart failure: Assessment of Reduction in Mortality and morbidity; APPROACH, Alberta Provincial Project for Outcome Assessment in Coronary Heart Disease; ASCEND-HF, Acute Study of Clinical Effectiveness of Nesiritide in Decompensated Heart Failure; ADHERE-EM, Acute Decompensated Heart Failure National Registry Emergency Module; PURSUIT- HFpEF, Prospective Multicenter Observational Study of Patients with Heart Failure with Preserved Ejection Fraction; I-PRESERVE, Irbesartan in Heart Failure with Preserved Ejection Fraction; TOPCAT, Aldosterone Antagonist Therapy for Adults With Heart Failure and Preserved Systolic Function; US, United States; BMI:, body mass index; HF, heart failure; DM, diabetes mellitus; AF, atrial fibrillation; MI, myocardial infarction; NYHA, New York Heart Association; LVEF, left ventricular ejection fraction; eGFR, estimated glomerular filtration rate; LDL-C, low-density lipoprotein cholesterol; BNP, brain natriuretic peptide; PCI, percutaneous coronary intervention; HP, hypertension; CHD, coronary heart disease; CAD, coronary artery disease; EF, ejection fraction; IHD, ischemic heart disease; PMI, pacemaker implantation; CABG, coronary artery bypass graft; ACE, angiotensin converting enzyme; ARB, angiotensin II receptor blockers; COPD, chronic obstructive pulmonary disease; SBP, systolic blood pressure; BMI, body mass index; DBP, Diastolic blood pressure; NT-proBNP, N-terminal pro-B-type natriuretic peptide; RAS, renin-angiotensin-system; MRA, mineralocorticoid receptor antagonist; ICD, implantable cardioverter defibrillator; CRT, cardiac resynchronization therapy; BUN, blood urea nitrogen; GFR, Glomerular filtration rate; JVP, jugular venous pressure; EMS, emergency medical services; TIA, transient ischemic attack; NR, not reported; HFpEF, heart failure preserved ejection fraction; HFmrEF, heart failure with mid-range ejection fraction.

Overall, this meta-analysis included 41,508 HFpEF patients, of whom 18,535 (44.65%) were men (ranging from 30.38% to 66.33%). The number of patients in each study ranged from 260 to 8,987, with males aged 66.3 to 79.9 and females aged 66 to 77. Four reports were from Asia (8, 17, 23, 25), two were from multiple centers, (16, 20) two were from Europe (9, 19), and six were from America (5, 18, 21, 22, 24, 26). Apart from 7 prospective cohort studies (8, 16, 19, 20, 21, 23, 25), the other 7 articles were retrospective cohort studies (5, 9, 17, 18, 22, 24, 26). This meta-analysis included 10,692 HFmrEF patients, of whom 6,607 (61.79%) were men (ranging from 60.66% to 73.20%). The patients in each study ranged from 758 to 9,225, with males aged 67.5 to 73 and females aged 70.3 to 77. Among the 14 studies that included patients with HFpEF, nine studies defined HFpEF as ejection fraction ≥50%, two studies defined EF ≥ 45%, and three studies defined EF > 40%. The EF of the HFmrEF definition was 40%–49% across all 3 studies (Table 1). The average age of HFpEF and HFmrEF (men vs. women); the mortality rate in men vs. women in HFpEF and HFmrEF patients are shown in Supplementary Table S4.

The adjustments for confounding factors varied greatly for all-cause mortality. Age, BMI/obesity, diabetes, and hypertension are considered the key variables affecting the prognosis of HF. One study did not adjust for age (5), four did not adjust for body mass index (BMI)/obesity (16, 18, 21, 26), six did not adjust for diabetes (8, 16, 17, 21, 25, 26), and seven did not adjust for hypertension (5, 8, 16, 17, 21, 25, 26). According to the NOS, all 14 studies (5, 8, 9, 16–26) with sex differences in outcomes in HFpEF and HFmrEF patients were rated as moderate to high quality, with scores ranging from 7 to 9 (Supplementary Table S5).

### 3.3 Sex differences in prognosis in HFpEF

#### 3.3.1 All-cause mortality and CV mortality

**Figure 2:**
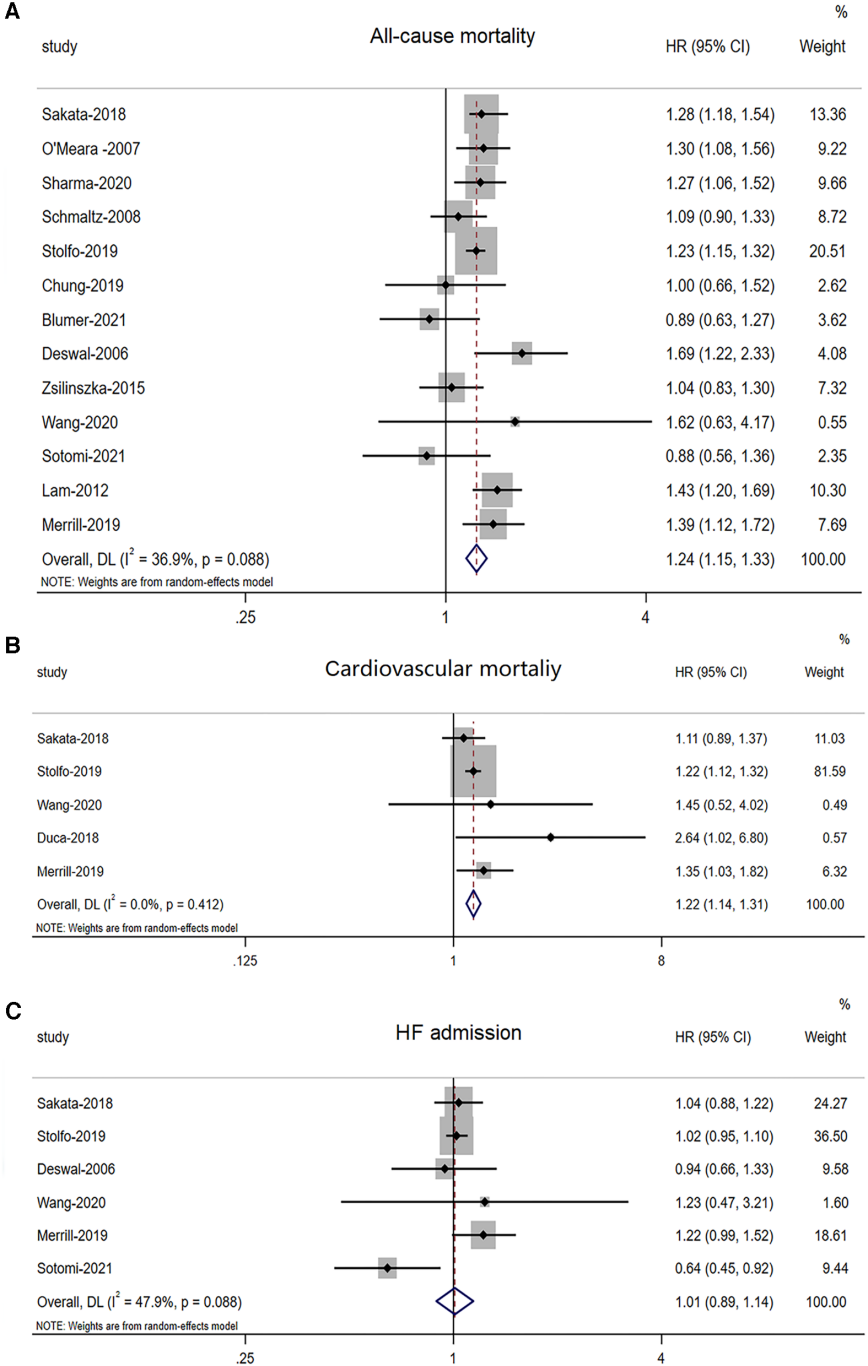
Forest plot of the association between sex differences and prognosis in patients with HFpEF. (**A**) Association between sex differences and all-cause mortality in patients with HFpEF. (**B**) Association between sex differences and CV mortality in patients with HFpEF. (**C**) Association between sex differences and HF admission in patients with HFpEF. In the forest plot, the diamond indicates the pooled estimate. Gray boxes are relative to study size, and the black vertical lines indicate 95% CIs around the effect size estimate. HFpEF, heart failure with preserved ejection fraction; CV, cardiovascular.

Thirteen studies (5, 8, 9, 16–18, 20–26) involving 41,248 HFpEF patients reported differences in all-cause mortality between males and females. There was a significant increase in all-cause mortality among male patients with HFmrEF (adjusted HR: 1.24, 95% CI: 1.15 to 1.33, –*P *< 0.0001) with evidence of heterogeneity (*I*^2 ^= 36.9%, *τ*^2 ^= 0.0053, *P *= 0.088) (Figure 2A).

Five studies (8, 9, 19, 24, 25) involving 15,670 HFpEF patients reported differences in CV mortality between males and females. There was a significant increase in CV mortality among male patients with HFmrEF. (adjusted HR: 1.22, 95% CI: 1.14 to 1.31, *P *< 0.0001) with no evidence of heterogeneity (*I*^2 ^< 0.001, *τ*^2 ^= 0.00, *P *= 0.412) (Figure 2B).

#### 3.3.2 HF admission

Six studies (8, 9, 18, 23–25) involving 17,008 HFpEF patients reported differences in HF admissions between males and females. However, there was no significant increase in HF admissions among male patients with HFmrEF (adjusted HR: 1.01, 95% CI: 0.89 to 1.14, *P *< 0.878) with evidence of heterogeneity (*I*^2 ^= 47.9%, *τ*^2 ^= 0.0095, *P *= 0.088) (Figure 2C).

### 3.4 Sex differences in prognosis in HFmrEF

#### 3.4.1 All-cause mortality and CV mortality

**Figure 3:**
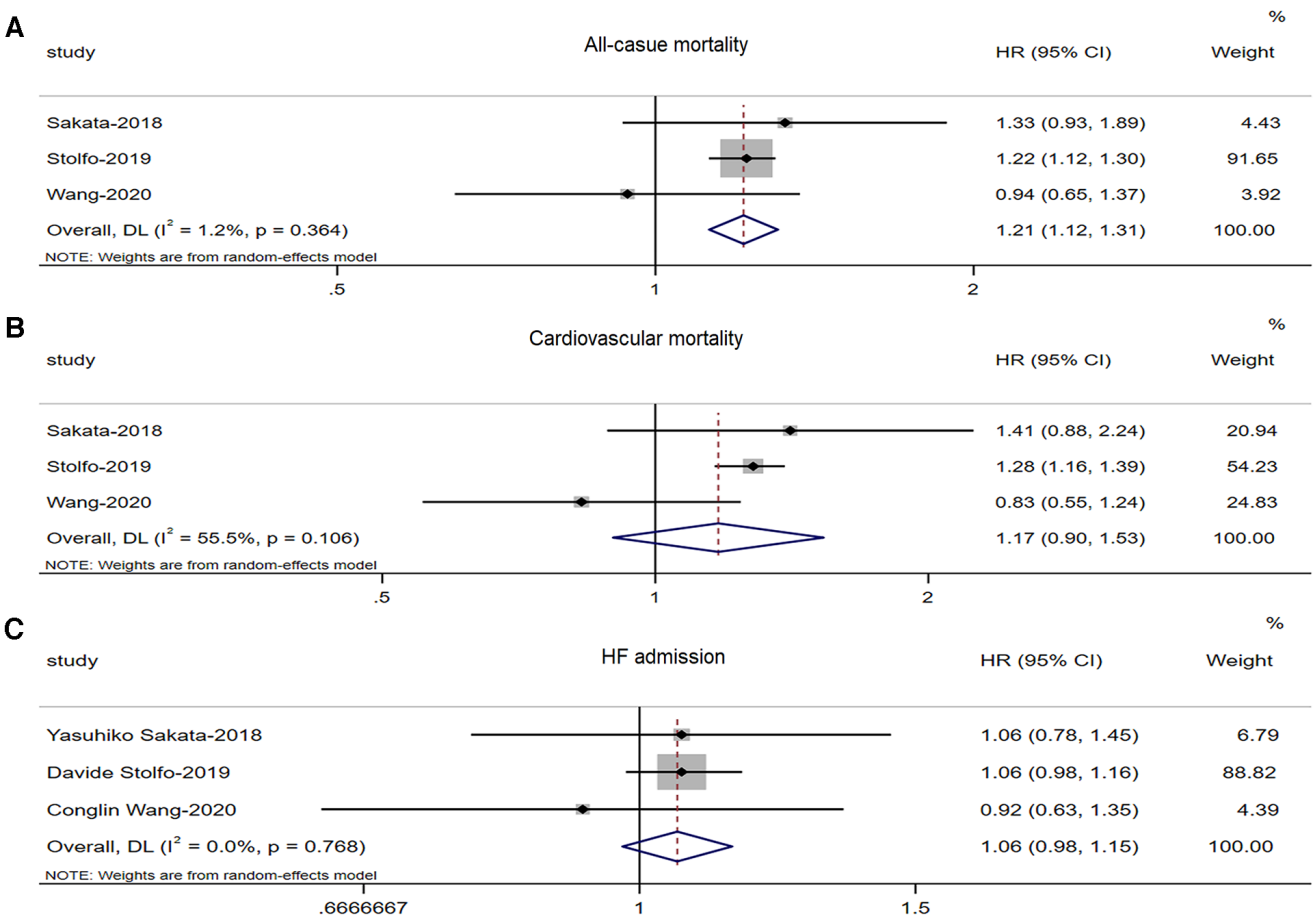
Forest plot of the association between sex differences and prognosis in patients with HFmrEF. (**A**) Association between sex differences and all-cause mortality in patients with HFmrEF. (**B**) Association between sex differences and CV mortality in patients with HFmrEF. (**C**) Association between sex differences and HF admission in patients with HFmrEF. HF, heart failure; HFmrEF, heart failure with mid-range ejection fraction; CV, cardiovascular.

Three studies (8, 9, 25) involving 10,692 HFmrEF patients reported differences in all-cause mortality between males and females. There was a significant increase in all-cause mortality among male patients with HFmrEF patients (adjusted HR: 1.21, 95% CI: 1.12 to 1.31, *P *< 0.0001) with no evidence of heterogeneity (*I*^2 ^= 1.2%, *τ*^2 ^= 0.0002, *P *= 0.364) (Figure 3A).

Three studies (8, 9, 25) involving 10,692 HFmrEF patients reported differences in CV mortality between males and females. There was no significant increase in CV mortality among male patients with HFmrEF (adjusted HR: 1.17, 95% CI: 0.90 to 1.53, *P *< 0.241) with no evidence of heterogeneity (*I*^2 ^= 55.5%, *τ*^2 ^= 0.032, *P *= 0.106) (Figure 3B).

#### 3.4.2 HF admission

Three studies (8, 9, 25) involving 10,692 HFmrEF patients reported differences in HF admissions between males and females. However, there was no significant increase in HF admissions among male patients with HFmrEF (adjusted HR: 1.06, 95% CI: 0.98 to 1.15, *P *< 0.146) with no evidence of heterogeneity (*I*^2 ^= 0%, *τ*^2 ^= 0.00, *P *= 0.768) (Figure 3C).

### 3.5 Subgroup and sensitivity analyses and publication bias

Subgroup analyses for all-cause death were performed according to study design, sample size, region, mean follow-up time, and adjustment for confounders (Table 2). In addition to the adjustment of the subgroup, the differences between the other subgroups were similar (*P* > 0.05). A stronger association was shown in the group adjusted for New York Heart Association (NYHA) and estimated glomerular filtration rate (eGFR)/glomerular filtration rate (GFR) (*P *< 0.1).

**Table 2:** Subgroup analysis of the impact of gender differences on all-cause mortality in patients with hFpEF.

Items		Number of studies	HR (95%CI)	*P*	*P* *_h (%)_	*P* **
Result of primary analysis		13	1.237 (1.152–1.329)	<0.001	36.9	–
Study design	Retrospective cohort	7	1.256 (1.157–1.364)	<0.001	28.2	0.474
	Prospective cohort	6	1.180 (1.015–1.371)	0.031	52.9	–
Sample size	<2,000	6	1.187 (0.936–1.504)	0.157	57.7	0.710
	≥2,000	7	1.243 (1.172–1.319)	<0.001	16.9	–
Region	Europe	1	1.235 (1.154–1.321)	<0.001	–	0.926
	America	6	1.254 (1.116–1.409)	<0.001	44.5	–
	Asia	4	1.167 (0.963–1.415)	0.115	22.1	–
	multicenter	2	1.158 (0.733–1.831)	0.530	82.4	–
Mean follow-up time(years)	≥3	8	1.280 (1.192–1.374)	<0.001	29.2	0.073
	<3	5	1.086 (0.922–1.280)	0.323	29.7	**-**
Adjustment for confounding factors						
Age	Yes	12	1.230 (1.137–1.330)	<0.001	41.5	0.598
	No	1	1.299 (1.077–1.566)	0.006	–	
BMI/obesity	Yes	9	1.263 (1.202–1.328)	<0.001	<0.001	0.358
	No	4	1.137 (0.912–1.416)	0.253	64.5	–
DM	Yes	7	1.304 (1.200–1.416)	<0.001	33.1	**0**.**043**
	No	6	1.125 (1.001–1.264)	0.049	23.2	
Ischemic heart disease	Yes	5	1.232 (1.087–1.397)	0.001	51.9	0.966
	No	8	1.237 (1.119–1.367)	<0.001	34.6	–
NYHA	Yes	6	1.303 (1.219–1.392)	<0.001	19.0	**0**.**006**
	No	7	1.101 (0.995–1.218)	0.062	<0.001	
Hypertension	Yes	6	1.311 (1.185–1.450)	<0.001	44.0	0.095
	No	7	1.160 (1.048–1.284)	0.004	23.1	
CHD	Yes	4	1.268 (1.086–1.481)	0.003	16.8	0.720
	No	9	1.228 (1.128–1.337)	<0.001	47.4	
Prior HF hospitalization	Yes	5	1.280 (1.159–1.413)	<0.001	30.4	0.406
	No	8	1.202 (1.076–1.343)	0.001	42.1	
AF	Yes	5	1.318 (1.195–1.454)	<0.001	11.6	0.147
	No	8	1.189 (1.078–1.312)	0.001	42.3	
eGFR/GFR	Yes	6	1.299 (1.217–1.388)	<0.001	18.8	**0**.**01**
	No	7	1.099 (0.985–1.226)	0.09	7.7	** **
Heart rate	Yes	9	1.277 (1.196–1.363)	<0.001	24.6	**0**.**013**
	No	4	1.030 (0.881–1.204)	0.714	<0.001	

HFpEF, heart failure with preserved ejection fraction; BMI, body mass index; DM, diabetes mellitus; NYHA, New York Heart Association; CHD, coronary heart disease; AF, atrial fibrillation; eGFR/GFR, estimated glomerular filtration rate/glomerular filtration rate.

* *P* for within-group heterogeneity.

** *P* for subgroup difference.

Egger's test (*P *= 0.632), Begg's test (*P *= 0.583) and funnel plots did not show statistically significant bias in potential publication. Sensitivity analyses confirmed the robustness performed by omitting each study or excluding studies with HFpEF with a definition of ejection fraction not less than 50% (Supplementary Figures S1–S2).

### 3.6 Quality of evidence assessment

Evidence was graded according to GRADE. The studies included in this meta-analysis were all reasonable, rigorous, and high-quality cohort studies. Finally, from the six included outcomes, the GRADE assessment showed moderate certainty for all-cause mortality, CV mortality, and HF admission in patients with HFpEF and HFmrEF (Supplementary Tables S6–S7).

## 4 Discussion

### 4.1 Major findings

In total, the study included 14 studies involving 52,200 patients with HFpEF (41,508) and HFmrEF (10,692). For HFpEF patients, men were significantly more likely than women to die from all causes and CVDs, but HF admission was not associated with sex differences; for HFmrEF patients, men were significantly more likely than women to die from all causes, but CV mortality and HF admission were not associated with sex differences. We systematically evaluated whether there are prognostic differences between men and women in patients with HFpEF and HFmrEF.

The sex difference in the prognosis of HFpEF and HPmrHF remains controversial. Previous studies reported a similar crude d mortality rate between sexes in patients with HF (27). Stolfo et al. showed that women had lower all-cause mortality (HR: 0.81, 95% CI: 0.76 to 0.87) and CV mortality (HR: 0.82, 95% CI: 0.76 to 0.89) than men among HFpEF patients, but HF admission did not decrease significantly (HR: 0.98, 95% CI: 0.91 to 1.05) (9). However, some studies have found no significant difference in the prognosis of HF by sex. For example, Blumer et al. showed that there was no prominent increase in all-cause mortality among male HFpEF patients. (HR: 1.12, 95% CI: 0.79 to 1.58) (16). Wang et al. showed that the prognosis was similar between men and women in HFpEF patients, including all-cause mortality (HR: 0.619, 95% CI: 0.240 to 1.593), CV mortality (HR: 0.690, 95% CI: 0.249 to 1.915) and HF admission (HR: 0.812, 95% CI: 0.312 to 2.114) (25). For patients with HFpEF, our results show that men were at greater risk for all-cause and CV death, while HF admission was similar to that in women. In general, statistical power was generally reduced when there were fewer studies included or insufficient follow-up. Therefore, the preliminary conclusion that sex has a prominent effect on the prognosis of HFpEF needs to be established by more large sample size and prospective studies.

For HFmrEF patients, our results showed that men were at greater risk for all-cause death, while CV death and HF admission were similar to those in women. Insufficient studies may have resulted in a nonsignificant increase in CV mortality (3 studies), and more prospective studies are needed to demonstrate the association between sex differences and CV mortality in HFmrEF.

Our results showed no statistically prominent differences in the outcomes of death from any causes or CVDs and HF admission between HFpEF and HFmrEF (all *P *> 0.1). Additionally, a large IPD meta-analysis consistently showed women had a lower age-adjusted all-cause mortality in either patient with HFpEF or HFrEF (interaction *p* value for EF group × sex = 0.72) (27), which reinforced our observation of better outcomes for women with HF compared with males, regardless of EF.

The etiology of HF is an important confounding factor. Studies have shown that men are more likely to suffer from ischemic heart disease (IHD) (28, 29). Our results showed that men had a higher all-cause mortality than women even after adjusting for IHD, and there was no difference between groups stratified by IHD (*P *= 0.966). These results suggested that IHD has no effect on death from any cause in HFpEF patients.

Diabetes is another vital potential mediator. Martínez's findings suggested that diabetes did not affect mortality for any cause (HR: 1.41, 95% CI: 1.35 to 1.47). It also found that among diabetic HFpEF patients, the HRs of men and women who died from any cause were not significantly different. However, among nondiabetic patients with HFpEF, men were more likely to die from any cause (27). The results from another study also suggested that sex did not influence mortality in HFpEF patients with diabetes but not in nondiabetic patients (18). Our subgroup analysis suggested a stronger relationship between men and all-cause death in subgroups with adjustment for diabetes mellitus (30) (*P *= 0.046). Subgroups stratified by adjustment for eGFR, NYHA, and heart rate had statistically prominent differences but not among subgroups adjusted for age, atrial fibrillation (AF), prior HF hospitalization, coronary heart disease (CHD), and obesity. Overall, these results suggested that the sex difference in prognosis in HFpEF could be partly explained by the kidney, diabetes, and severity of HF rather than IHD, AF, hypertension, age, and obesity.

### 4.2 Comparison with previous studies

The prior meta-analysis conducted by Manuel et al. showed that being male is independently associated with an increased risk of all-cause mortality in patients with both HFrEF and HFpEF (27). Furthermore, our study has revealed a link between male sex and cardiovascular mortality in HFpEF.

HFpEF is increasingly recognized as a syndrome with diverse phenotypes and various comorbidities. Notably, cardiac-related deaths account for only 27% of all-cause mortality in HFpEF patients, as opposed to 65% in HFrEF. This finding suggests that the disparity in mortality between sexs in HFpEF can be partly attributed to cardiac factors. Additionally, there has been limited exploration of sex-related differences and outcomes in HFmrEF. Our research has demonstrated that women tend to have better survival rates among patients with HFmrEF, underscoring the persistence of sex-related variations in prognosis regardless of ejection fraction.

### 4.3 Potential mechanism

The underlying mechanism of sex differences related to the prognosis of HF is unclear. In general, women with typical HFpEF have more complications (31), with hypertension and diabetes being the main cardiovascular risk factors associated with HFpEF (29). Men are more likely to suffer from HFrEF and HFmrEF (31), and ischemic cardiomyopathy is more common as a cause of HF (28, 29).

The reason why women have a higher survival rate than men may be that they have better heart function and less ischemic cardiomyopathy (28, 32). Studies have shown that estrogen, the main sex hormone in women, plays a crucial role in heart health. In addition to protecting the heart from cardiomyocyte hypertrophy and apoptosis, myocardial infarction size, and arrhythmia, estrogen reduces ischemic-reperfusion injury (IRI) (33–37). In addition, estrogen can regulate some risk factors for CHD, such as hypertension and hyperlipidemia, by reducing the vasoconstrictor endothelin and increasing the activity of lipoprotein lipase to prevent CHD and HF (38–41).

### 4.4 Clinical implications

HF treatment is aimed at reducing symptoms, improving survival, enhancing physical activity, and making patients live better (42). Treatment of patients with systolic dysfunction aims to reduce elevated filling pressures, decrease neurohormonal levels, and increase cardiac output. In patients with diastolic dysfunction, the main purpose of treatment is to improve ventricular relaxation and filling and reduce preload (30). However, there is no model for classifying treatment by sex. Our comprehensive study revealed that male patients with HFpEF and HFmrEF have a worse prognosis. Consistent with prior studies, women generally exhibited better prognoses than men, irrespective of EF. Consequently, further research is essential to better understand the observed sex difference in prognosis in patients with HFpEF and HFmrEF and how both pathophysiology and treatments contribute to this.

### 4.5 Study limitations

The present systematic reviews and meta-analyses have several limitations. First, half of the retrospective cohorts were included in the study. However, the subgroup analysis of prospective and retrospective studies was consistent, showing the robustness of the present study. Second, the EF of patients with HFpEF across the included studies was not uniform, and the EF range of some patients with HFpEF overlapped the EF range of patients with HFmrEF (Table 1). However, sensitivity analysis of all studies with an ejection fraction of no less than 50% still showed that our results were stable and reliable (Supplementary Figure S1B). There was variability in the HFpEF definition (with a cutoff of 40%, 45%, or 50%), resulting in some studies including HFmrEF patients as HFpEF, which is inconsistent with the latest HF Universal definition. These cutoffs might have over/underestimated the current findings. This constitutes one of the significant limitations of the present study and may limit its generalizability. Third, the number of included studies was limited, and more studies were included to prove the reliability of the conclusions. The other limitation is an inherent restriction of observational studies and the potential for some confounders not adjusted for- this should be included in the limitations. Last, patients included in these studies might have been categorized into HF phenotypes based on only one single measure of EF. In addition to the variability of study definitions, the variability in clinical assessment might also contribute to patients' misclassifications. Despite these limitations, it is important to consider sex differences in clinical settings, and our study provides valuable information for the design and analysis of clinical trials and animal studies related to HFmrEF and HFpEF, which are two types of HF with limited treatment options.

## 5 Conclusions

Among HFpEF patients, men were prominently more likely than women to die from all causes and CVDs, but their HF admissions were similar; among HFmrEF patients, men were prominently more likely than women to die from all causes, but their CV mortality and HF admissions were similar. Overall, women with HF may have better survival than men, regardless of EF.

## Data Availability

The original contributions presented in the study are included in the article/Supplementary Material, further inquiries can be directed to the corresponding author.
