# Supplementary material for: Sex differences in mortality and hospitalization in heart failure with preserved and mid-range ejection fraction: a systematic review and meta-analysis of cohort studies
Source: Front Cardiovasc Med. 2024 Jan 5;10:1257335. doi: 10.3389/fcvm.2023.1257335 (PMC10796792; doi:10.3389/fcvm.2023.1257335)

**Supplementary Table 1. PRISMA Checklist.**

| **Section/topic** | **#** | **Checklist item** | **Reported on page #** |
| --- | --- | --- | --- |
| **TITLE** | | |  |
| Title | 1 | Identify the report as a systematic review, meta-analysis, or both. |  |
| **ABSTRACT** | | |  |
| Structured summary | 2 | Provide a structured summary including, as applicable: background; objectives; data sources; study eligibility criteria, participants, and interventions; study appraisal and synthesis methods; results; limitations; conclusions and implications of key findings; systematic review registration number. |  |
| **INTRODUCTION** | | |  |
| Rationale | 3 | Describe the rationale for the review in the context of what is already known. |  |
| Objectives | 4 | Provide an explicit statement of questions being addressed with reference to participants, interventions, comparisons, outcomes, and study design (PICOS). |  |
| **METHODS** | | |  |
| Protocol and registration | 5 | Indicate if a review protocol exists, if and where it can be accessed (e.g., Web address), and, if available, provide registration information including registration number. |  |
| Eligibility criteria | 6 | Specify study s (e.g., PICOS, length of follow-up) and report s (e.g., years considered, language, publication status) used as criteria for eligibility, giving rationale. |  |
| Information sources | 7 | Describe all information sources (e.g., databases with dates of coverage, contact with study authors to identify additional studies) in the search and date last searched. |  |
| Search | 8 | Present full electronic search strategy for at least one database, including any limits used, such that it could be repeated. |  |
| Study selection | 9 | State the process for selecting studies (i.e., screening, eligibility, included in systematic review, and, if applicable, included in the meta-analysis). |  |
| Data collection process | 10 | Describe method of data extraction from reports (e.g., piloted forms, independently, in duplicate) and any processes for obtaining and confirming data from investigators. |  |
| Data items | 11 | List and define all variables for which data were sought (e.g., PICOS, funding sources) and any assumptions and simplifications made. |  |
| Risk of bias in individual studies | 12 | Describe methods used for assessing risk of bias of individual studies (including specification of whether this was done at the study or outcome level), and how this information is to be used in any data synthesis. |  |
| Summary measures | 13 | State the principal summary measures (e.g., risk ratio, in means). |  |
| Synthesis of results | 14 | Describe the methods of handling data and combining results of studies, if done, including measures of consistency (e.g., I^2^) for each meta-analysis. |  |
| Risk of bias across studies | 15 | Specify any assessment of risk of bias that may affect the cumulative evidence (e.g., publication bias, selective reporting within studies). |  |
| Additional analyses | 16 | Describe methods of additional analyses (e.g., sensitivity or subgroup analyses, meta-regression), if done, indicating which were pre-specified. |  |
| **RESULTS** | | |  |
| Study selection | 17 | Give numbers of studies screened, assessed for eligibility, and included in the review, with reasons for exclusions at each stage, ideally with a flow diagram. |  |
| Study s | 18 | For each study, present s for which data were extracted (e.g., study size, PICOS, follow-up period) and provide the citations. |  |
| Risk of bias within studies | 19 | Present data on risk of bias of each study and, if available, any outcome level assessment (see item 12). |  |
| Results of individual studies | 20 | For all outcomes considered (benefits or harms), present, for each study: (a) simple summary data for each intervention group (b) effect estimates and confidence intervals, ideally with a forest plot. |  |
| Synthesis of results | 21 | Present results of each meta-analysis done, including confidence intervals and measures of consistency. |  |
| Risk of bias across studies | 22 | Present results of any assessment of risk of bias across studies (see Item 15). |  |
| Additional analysis | 23 | Give results of additional analyses, if done (e.g., sensitivity or subgroup analyses, meta-regression [see Item 16]). |  |
| **DISCUSSION** | | |  |
| Summary of evidence | 24 | Summarize the main findings including the strength of evidence for each main outcome; consider their relevance to key groups (e.g., healthcare providers, users, and policy makers). |  |
| Limitations | 25 | Discuss limitations at study and outcome level (e.g., risk of bias), and at review-level (e.g., incomplete retrieval of identified research, reporting bias). |  |
| Conclusions | 26 | Provide a general interpretation of the results in the context of other evidence, and implications for future research. |  |
| **FUNDING** | | |  |
| Funding | 27 | Describe sources of funding for the systematic review and other support (e.g., supply of data); role of funders for the systematic review. |  |

*From:*  Moher D, Liberati A, Tetzlaff J, Altman DG, The PRISMA Group (2009). Preferred Reporting Items for Systematic Reviews and Meta-Analyses: The PRISMA Statement. PLoS Med 6(7): e1000097. doi:10.1371/journal.pmed1000097

For more information, visit: **www.prisma-statement.org**

**Supplementary Table 2. Search strategy**

**PubMed database:**

| Search | Query |
| --- | --- |
| #1 | (("Sex "[Mesh]) OR (gender)) |
| #2 | ((((((Heart failure) AND (mid-range ejection fraction)) OR (Heart failure preserved ejection fraction)) OR (HFpEF)) OR (HFmrEF)) OR (HF)) OR (" Heart Failure "[Mesh]) |
| #3 | #1 AND #2  ((("Sex s"[Mesh]) OR (gender)) AND (((((((Heart failure) AND (mid-range ejection fraction)) OR (Heart failure preserved ejection fraction)) OR (HFpEF)) OR (HFmrEF)) OR (HF[Title/Abstract])) OR ("Heart Failure"[Mesh])) |

**Embase database:**

| Search | Query |
| --- | --- |
| #1 | 'sex'/exp OR gender |
| #2 | sex':ab,ti OR 'sex ':ab,ti OR 'dimorphism':ab,ti OR 'dimorphism, sexual':ab,ti OR 'dimorphisms':ab,ti OR 'gender s':ab,ti OR 'gender ':ab,ti OR 'sex dimorphism':ab,ti OR 'dimorphism, sex':ab,ti OR 'sex dimorphisms':ab,ti OR 'gender s':ab,ti OR , gender':ab,ti OR 'gender ':ab,ti OR 'gender dimorphism':ab,ti OR 'dimorphism, gender':ab,ti OR 'gender dimorphisms':ab,ti OR 'sex s':ab,ti OR ', sex':ab,ti OR 'sex ':ab,ti OR 'dichromatism':ab,ti OR 'dichromatism, sexual':ab,ti OR 'dichromatisms, sexual':ab,ti OR 'dichromatisms':ab,ti |
| #3 | 'hfmref': ab,ti OR 'hfpef':ab,ti OR 'heart failure preserved ejection fraction':ab,ti OR 'heart failure with mild ejection fraction': ab, ti OR 'heart failure with intermediate ejection fraction': ab, ti |
| #4 | #1 OR #2 |
| #5 | #3 AND #4 |

**Cochrane library:**

| Search | Query |
| --- | --- |
| #1 | MeSH descriptor: [ Sex, gender] explode all tree |
| #2 | (Sex or Sex or Dimorphism or Dimorphism, or Dimorphisms or Gender s or Gender or Sex Dimorphism or Dimorphism, Sex or Sex Dimorphisms or Gender s or , Gender or Gender or Gender Dimorphism or Dimorphism, Gender or Gender Dimorphisms or Sex s or , Sex or Sex or Dichromatism or Dichromatism, or Dichromatisms, or Dichromatisms): ti,ab,kw |
| #3 | MeSH descriptor: [Heart failure] explode all tree |
| #4 | (Heart failure with mid-range ejection OR Heart failure with mild reduced ejection fraction OR Heart failure with intermediate ejection fraction OR HFmrEF OR HFpEF OR Heart failure preserved ejection fraction): ti,ab,kw |
| #5 | #1 OR #2 |
| #6 | #3 OR #4 |
| #7 | #5 AND #6 |

**Supplementary Table 3. Studies excluded(n=24) with reasons**

| Studies excluded | Reasons (according to PICOS) |
| --- | --- |
| Louise Y Sun-2020[1] | No classification of heart failure types |
| Julio Nu´ ~nez-2021[2] | Unrelated to the prognosis of patients with HFpEF or HFmrEF |
| Yasuki Nakada-2016[3] | No extractable related data |
| Claire A. Lawson-2020[4] | Unrelated to the prognosis of patients with HFpEF or HFmrEF |
| Anubha Agarwal-2021[5] | No classification of heart failure types |
| Gianluigi Savarese-2018[6] | Reviews |
| Yasuhiko Sakata-2014[7] | No classification of heart failure types |
| John T. Parissis-2013[8] | No classification of heart failure types |
| Dan Rusinaru -2009[9] | No extractable related data |
| ROBERT KLEMPFNER-2014[10] | No classification of heart failure types |
| Michael C. Honigberg-2020[11] | Unrelated to the prognosis of patients with HFpEF or HFmrEF |
| Muhammed T. Gürgöze-2021[12] | Meta-analyses |
| SOFIE A. GEV AERT-2014[13] | No classification of heart failure types |
| Mary Roberts Davis-2021[14] | Meta-analyses |
| Louise Y. Sun-2018[15] | No classification of heart failure types |
| Youn-Jung Son-2021[16] | No extractable related data |
| FA´TIMA RODRIGUEZ-2013[17] | No extractable related data |
| Sibille Lejeune-2020[18] | Conference Abstract |
| Tabassome Simon-2001[19] | No classification of heart failure types |
| U Ljung Faxen-2017[20] | Conference Abstract |
| Okechukwu S. Ogah-2015[21] | No classification of heart failure types |
| Katsuya Kajimoto-2017[22] | No extractable related data |
| Shinichiro Suna-2014[23] | No classification of heart failure types |
| Wilfried Mullens-2008[24] | No classification of heart failure types |

**Supplementary Table 4. The average age and mortality rate in men vs. women in HFpEF and HFmrEF patients.**

|  | HFpEF | | | |  |  | HFmrEF | | | |
| --- | --- | --- | --- | --- | --- | --- | --- | --- | --- | --- |
|  | Age(y) | | Death/Sample,  Mortality (%) | |  |  | Age(y) | | Death/Sample,  Mortality (%) | |
| Author, year | Men | Women | Men | Women |  |  | Men | Women | Men | Women |
| Sharma et al, 2020 | 75.6 | 78.1 | 1085/3153  34.4 | 1914/5834  32.8 |  |  | NA | NA | NA | NA |
| Deswal et al, 2006 | 67 | 70 | 91/378  24 | 85/341  25 |  |  | NA | NA | NA | NA |
| Lam et al,2012 | 71 | 72 | 434/1637  26.5 | 447/2491  17.9 |  |  | NA | NA | NA | NA |
| Wang et al, 2020 | 70.73 | 73.59 | NA | NA |  |  | 69.37 | 72.8 | NA | NA |
| Stolfo et al, 2019 | 75 | 79 | 1888/4516  41.8 | 2373/5441  43.6 |  |  | 73 | 77 | 2009/5596  35.9 | 1459/3629  40.2 |
| O'Meara et al, 2007 | NA | NA | 281/1811  15.5 | 200/1212  16.5 |  |  | NA | NA | NA | NA |
| Duca et al, 2018 | 72 | 73 | 15/79  18.99 | 30/181  16.57 |  |  | NA | NA | NA | NA |
| Schmaltz et al, 2008 | 66.3 | 69 | NA | NA |  |  | NA | NA | NA | NA |
| Chung et al, 2019 | 67.3 | 71.1 | 81/256  31.6 | 145/471  30.8 |  |  | 62.8 | 69.1 | 261/950  27.5 | 206/734  28.1 |
| Merrill et al, 2019 | 70 | 72.1 | 107/441  24 | 98/440  22 |  |  | NA | NA | NA | NA |
| Zsilinszka et al, 2015 | 79.9 | 82.4 | 336/1353  24.8 | 666/2808  23.7 |  |  | NA | NA | NA | NA |
| Sakata et al, 2018 | 68.3 | 71.6 | 620/2068  30 | 355/1124  31.6 |  |  | 67.5 | 70.3 | 168/519  32.4 | 63/190  33.2 |
| Sotomi et al, 2021 | 79.75 | 82.23 | 50/389  12.8 | 62/481  12.8 |  |  | NA | NA | NA | NA |

**HFpEF=heart failure with preserved ejection fraction; HFmrEF=heart failure with intermediate ejection fraction, NA= not reported**

**Supplementary Table 5. Quality assessment of the included studies by Newcastle–Ottawa scale.**

| Study | Selection | | | | Comparability | Outcome | | | Total |
| --- | --- | --- | --- | --- | --- | --- | --- | --- | --- |
|  | Exposed cohort | Nonexposed cohort | Ascertainment of exposure | Outcome of interest |  | Assessment of outcome | Length of follow-up | Adequacy of follow-up |  |
| Yasuhiko Sakata-2018 | * | * | * | * | ** | * | * | * | 9 |
| O'Meara Eileen-2007 | * | * |  | * | ** | * | * | * | 8 |
| Kavita Sharma-2020 | * | * | * | * | ** | * |  | * | 8 |
| Heidi N. Schmaltz-2008 | * | * | * | * | * | * | * | * | 8 |
| Davide Stolfo-2019 | * | * | * | * | ** | * | * | * | 9 |
| Jaehoon Chung-2019 | * | * |  | * | ** | * | * | * | 8 |
| Vanessa Blumer-2021 | * | * | * | * | * | * |  | * | 7 |
| Anita Deswal-2006 | * | * | * | * | ** | * | * | * | 7 |
| Reka Zsilinszka-2015 | * | * | * | * | * | * |  | * | 7 |
| Conglin Wang-2020 | * | * | * | * | ** | * |  | * | 8 |
| Yohei Sotomi-2021 | * | * | * | * | ** | * |  | * | 8 |
| Carolyn S.P. Lam-2012 | * | * | * |  | ** | * | * | * | 8 |
| Franz Duca-2018 | * | * | * | * | ** | * |  | * | 8 |
| Miranda Merrill-2019 | * | * | * | * | * | * | * | * | 9 |

Comparability: the most important factor is age; Other important confounders is body mass index (BMI)/obesity

Length of follow-up: ≥3years

**Supplementary Table 6. GRADE evidence profile for the all-cause mortality, CV mortality, and HF admission in patients with HFpEF.**

| **Certainty assessment** | | | | | | | **№ of patients** | | **Effect** | | **Certainty** | **Importance** |
| --- | --- | --- | --- | --- | --- | --- | --- | --- | --- | --- | --- | --- |
| **№ of studies** | **Study design** | **Risk of bias** | **Inconsistency** | **Indirectness** | **Imprecision** | **Other considerations** | **men in prognosis among patients with HFpEF** | **women in prognosis among patients with HFpEF** | **Relative (95% CI)** | **Absolute (95% CI)** |  |  |
| **All-cause mortality** | | | | | | | | | | | | |
| 13 | observational studies | not serious | not serious | not serious | not serious | none | 0/18456 (0.0%) | -/22792 | **HR 1.24** (1.15 to 1.33) | **-- per 1,000** (from -- to --) | ⨁⨁⨁◯  Moderate | CRITICAL |
| **CV mortality** | | | | | | | | | | | | |
| 5 | observational studies | not serious | not serious | not serious | not serious | none | 1380/7881 (17.5%) | 1695/7798 (21.7%) | **HR 1.22** (1.14 to 1.31) | **41 more per 1,000** (from 26 more to 57 more) | ⨁⨁⨁◯  Moderate | CRITICAL |
| **HF admission** | | | | | | | | | | | | |
| 6 | observational studies | not serious | not serious | not serious | not serious | none | -/8569 | -/8439 | **HR 1.01** (0.89 to 1.14) | **-- per 1,000** (from -- to --) | ⨁⨁⨁◯  Moderate | CRITICAL |

**Abbreviation:** GRADE= Grading of Recommendations Assessment, Development, and Evaluation; CV= cardiovascular; HF=heart failure; HFpEF=heart failure with preserved ejection fraction; CI=confidence interval; HR=hazard ratio.

**Supplementary Table 7. GRADE evidence profile for the all-cause mortality, CV mortality, and HF admission in patients with HFmrEF.**

| **Certainty assessment** | | | | | | | **№ of patients** | | **Effect** | | **Certainty** | **Importance** |
| --- | --- | --- | --- | --- | --- | --- | --- | --- | --- | --- | --- | --- |
| **№ of studies** | **Study design** | **Risk of bias** | **Inconsistency** | **Indirectness** | **Imprecision** | **Other considerations** | **men in prognosis among patients with HFmrEF** | **women in prognosis among patients with HFmrEF** | **Relative (95% CI)** | **Absolute (95% CI)** |  |  |
| **All-cause mortality** | | | | | | | | | | | | |
| 3 | observational studies | not serious | not serious | not serious | not serious | none | 2288/6607 (34.6%) | 1594/4085 (39.0%) | **HR 1.21** (1.12 to 1.31) | **60 more per 1,000** (from 35 more to 87 more) | ⨁⨁⨁◯  Moderate | CRITICAL |
| **CV mortality** | | | | | | | | | | | | |
| 3 | observational studies | not serious | not serious | not serious | not serious | none | 1400/6607 (21.2%) | 1003/4085 (24.6%) | **HR 0.82** (0.69 to 0.97) | **39 fewer per 1,000** (from 69 fewer to 6 fewer) | ⨁⨁⨁◯  Moderate | CRITICAL |
| **HF admission** | | | | | | | | | | | | |
| 3 | observational studies | not serious | not serious | not serious | not serious | none | -/6607 | -/4085 | **HR 0.95** (0.88 to 1.04) | **-- per 1,000** (from -- to --) | ⨁⨁⨁◯  Moderate | CRITICAL |

**Abbreviation:** GRADE= Grading of Recommendations Assessment, Development, and Evaluation; CV= cardiovascular; HF=heart failure; HFmrEF=heart failure with mid-ranged ejection fraction; CI=confidence interval; HR=hazard ratio.

**Supplementary Fig. 1 Sensitivity analysis of included studies that reported the association between gender s and all-cause mortality in patients with HFpEF**

A: HFpEF with an ejection fraction of not less than 40%; B: HFpEF with an ejection fraction of not less than 50%)

**
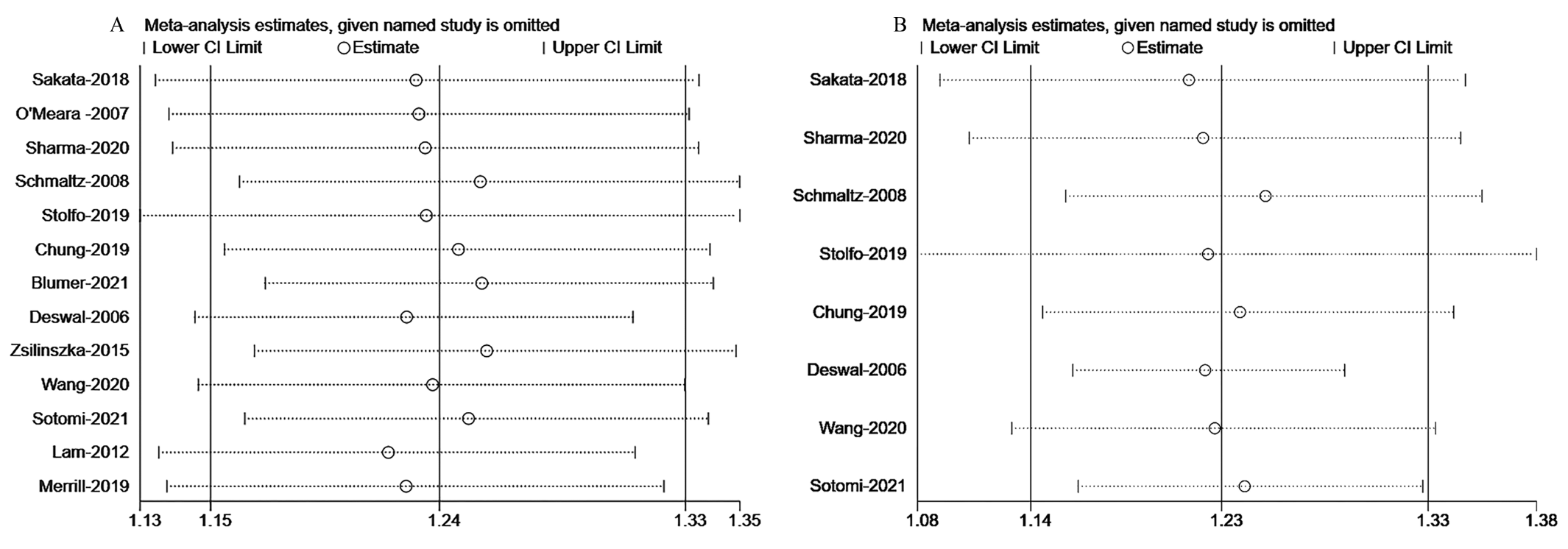
**

**Supplementary Fig. 2 Publication bias detected by funnel plot, Egger’s test and Begg’s test for the association between gender s and all-cause mortality in patients with HFpEF**

A: Funnel plot; B: Egger’s test; C: begg’s test


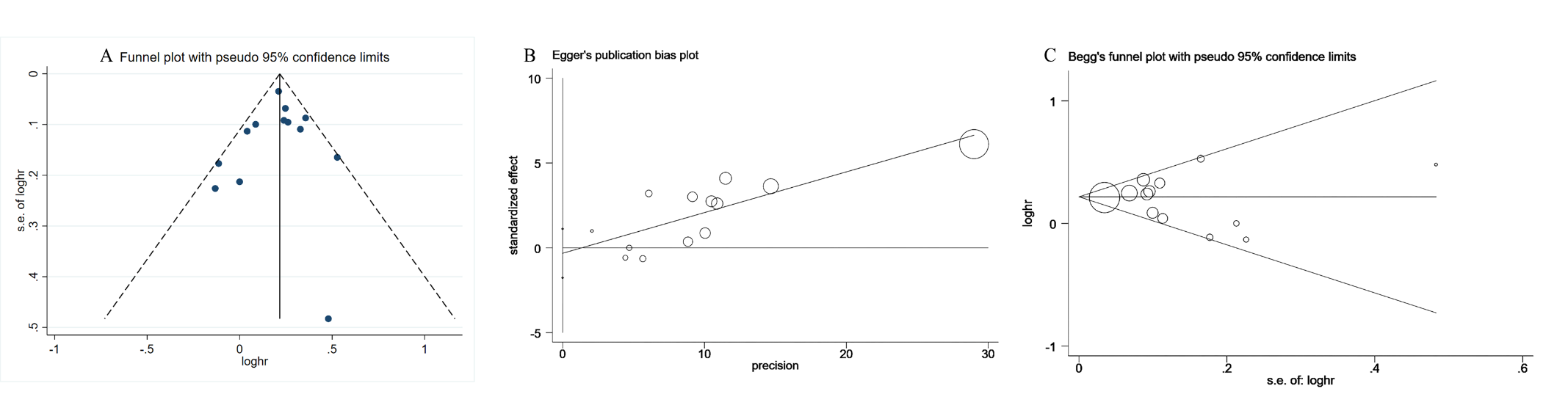

Supplement: Supplementary file 1 [file Datasheet1.docx]
